# Error in Author Initials

**DOI:** 10.1001/jamahealthforum.2025.3831

**Published:** 2025-08-22

The Research Letter titled “Projected Health System and Economic Impacts of 2025 Medicaid Policy Proposals,”^1^ which published July 16, 2025, misstated 2 authors’ middle initials in the byline and Corresponding Author section. The first author’s name is Sanjay Basu, and the second author’s name is Sadiq Y. Patel. This article has been corrected.
